# CydDC-mediated reductant export in *Escherichia coli* controls the transcriptional wiring of energy metabolism and combats nitrosative stress

**DOI:** 10.1042/BJ20150536

**Published:** 2016-03-10

**Authors:** Louise V. Holyoake, Stuart Hunt, Guido Sanguinetti, Gregory M. Cook, Mark J. Howard, Michelle L. Rowe, Robert K. Poole, Mark Shepherd

**Affiliations:** * School of Biosciences, University of Kent, Canterbury CT2 7NJ, U.K.; † Department of Molecular Biology and Biotechnology, The University of Sheffield, Sheffield S10 2TN, U.K.; ‡ School of Informatics, The University of Edinburgh, Informatics Forum, 10 Crichton Street, Edinburgh EH8 9AB, U.K.; § Department of Microbiology and Immunology, University of Otago, P.O. Box 56, 720 Cumberland Street, Dunedin 9054, New Zealand

**Keywords:** cysteine, cytochrome, glutathione, nitric oxide, nitrosative stress, redox regulation

## Abstract

The glutathione/cysteine exporter CydDC maintains redox balance in *Escherichia coli*. A *cydD* mutant strain was used to probe the influence of CydDC upon reduced thiol export, gene expression, metabolic perturbations, intracellular pH homoeostasis and tolerance to nitric oxide (NO). Loss of CydDC was found to decrease extracytoplasmic thiol levels, whereas overexpression diminished the cytoplasmic thiol content. Transcriptomic analysis revealed a dramatic up-regulation of protein chaperones, protein degradation (via phenylpropionate/phenylacetate catabolism), β-oxidation of fatty acids and genes involved in nitrate/nitrite reduction. ^1^H NMR metabolomics revealed elevated methionine and betaine and diminished acetate and NAD^+^ in *cydD* cells, which was consistent with the transcriptomics-based metabolic model. The growth rate and ΔpH, however, were unaffected, although the *cydD* strain did exhibit sensitivity to the NO-releasing compound NOC-12. These observations are consistent with the hypothesis that the loss of CydDC-mediated reductant export promotes protein misfolding, adaptations to energy metabolism and sensitivity to NO. The addition of both glutathione and cysteine to the medium was found to complement the loss of *bd*-type cytochrome synthesis in a *cydD* strain (a key component of the pleiotropic *cydDC* phenotype), providing the first direct evidence that CydDC substrates are able to restore the correct assembly of this respiratory oxidase. These data provide an insight into the metabolic flexibility of *E. coli*, highlight the importance of bacterial redox homoeostasis during nitrosative stress, and report for the first time the ability of periplasmic low molecular weight thiols to restore haem incorporation into a cytochrome complex.

## Abstract (precis)

The CydDC ABC transporter of *E. coli* exports glutathione and cysteine. Loss of *cydDC* elicits adaptations in energy metabolism and induces sensitivity to NO. CydDC therefore has a likely role in growth and survival during infection.

## INTRODUCTION

Early phenotyping of *Escherichia coli cydDC* mutants reported that this locus was required for the correct assembly of cytochrome *bd*-type quinol oxidases [1,2]. Based both on the observed gene structure and deduced protein sequences, CydDC was postulated to form a heterodimeric ABC transporter with two transmembrane domains, each comprising six α-helices, and two nucleotide binding domains [3]. The periplasm of strains lacking a functional CydDC were reported to be ‘over-oxidized’ [4], and subsequent functional characterization revealed that CydDC exports both reduced glutathione (GSH) [5] and cysteine [6] to the periplasm.

The phenotype of the *cydD* mutant strain is pleiotropic, exhibiting sensitivity to benzylpenicillin and dithiothreitol, loss of motility and the absence of holocytochrome *c*. These characteristics are reminiscent of *E. coli dsb* mutants [7], implicating defective disulfide bond formation/isomerization in the manifestation of these traits in *cydDC* mutant strains. These phenotypes, together with the loss of periplasmic cytochrome *b*_562_ (a typical *cydDC* phenotype), can all be corrected by the addition of cysteine or GSH to the growth medium [5,6,8], although the addition of GSH or cysteine alone cannot restore the assembly of *bd*-type quinol oxidases [5,6]. These observations point towards a role for CydDC in maintaining the periplasm at an optimum redox poise that is required for correct disulfide bond formation, and for the incorporation of haem cofactors into respiratory complexes and periplasmic hemoproteins. The involvement of CydDC-mediated glutathione/cysteine translocation in hemoprotein assembly is supported by the observation that CydDC overexpression under anaerobic conditions leads to the accumulation of a novel haem compound P-574 associated with the inner membrane [9]. Additionally, anaerobically grown *E. coli* overexpressing CydDC was found to contain a haem-bound form of NikA, a periplasmic nickel chaperone [10]. More recently, electron cryomicroscopy has confirmed a heterodimeric structure for CydDC, spectroscopic analyses reveal that haem *b* is bound to the purified complex, and kinetic assays demonstrate a stimulation of ATPase activity upon addition of haem and various thiol compounds [11]. Although assigning a role for haem transport is tempting for CydDC, previous investigations indicate that this is not a physiological role for this transporter [12], indicating a likely role for the haem cofactor in redox sensing.

Clearly, the contribution of CydDC to the maintenance of redox homoeostasis has an intricate relationship with cellular metabolism. Herein, we probe the response of *E. coli* to changing internal redox poise through the transcriptomic and physiological analysis of a *cydD* mutant strain.

## EXPERIMENTAL PROCEDURES

### Bacterial strains and growth conditions

The bacterial strains used in this work are from the Keio collection [13]. The wild-type strain and the isogenic *cydD* strain have a BW25113 [14] background. Starter cultures (10 ml) were grown to stationary phase in LB, and 0.5 ml was used to inoculate 50 ml of defined medium [15] supplemented with 0.1% (w/v) casamino acids. For the microarray experiments, cells were grown in 250 ml Klett flasks (sidearm) at 37°C and 180 rpm in triplicate, and culture turbidities were measured in Klett flasks using a Klett-Summerson colorimeter (red filter). Kanamycin (50 μg/ml) was included where appropriate, and glycerol (54 mM) was used as a carbon source. A typical *cydD* phenotype, the absence of a cytochrome *d* spectroscopic signal [2], was confirmed for the BW25113-*cydD* strain. For the growth curves in the presence of nitric oxide (NO)-releasing compounds, a Fluostar Omega plate reader (BMG Labtech) was used to measure OD_600_ for at least four repeats of each condition. The same growth medium was used as above, and NOC-12 was dissolved in sterile 80 mM sodium phosphate buffer (pH 8.0) immediately before use and added to the microplate wells at the time of inoculation. Growth rates were calculated from 2 h after the end of the lag phase for the [NOC-12]=0 data, which was defined as the intercept on the time axis created by the regression line for the maximal growth rate.

### Whole-cell absorption spectroscopy

To generate microaerobic conditions that enhance the assembly of *bd*-type cytochromes, *E. coli* cultures (100 ml) were grown overnight in LB medium in 250 ml conical flasks at 37°C and 150 rpm. Cells were harvested and CO-difference spectra were recorded on whole cells as previously described [16].

### Quantification of reduced thiol concentration

Cells were grown exactly as for the microarray experiments and growth medium was isolated from exponentially-growing cells via centrifugation of cultures (2342 ***g***, 4°C, 10 min). Spheroplasts were prepared using a modified osmotic shock procedure [17]. In brief, 5 ml of exponentially-growing cells were conditioned for osmotic shock via addition of NaCl and Tris/HCl buffer (pH 7.3) to give final concentrations of 27 mM each. Cells were harvested (2342 ***g***, 20 min, 20°C), suspended in 35 μl of supernatant from the first centrifugation and supplemented with 35 μl of a 40% (w/v) sucrose solution containing 33 mM Tris/HCl pH 7.3 and 2 mM EDTA. The cell suspension was then incubated at room temperature (20 min) and harvested by centrifugation (3024 ***g***, 20 min 20°C). Cells were then suspended in ice cold water (250 μl) and left on ice for 45 s before MgCl_2_ was added (final concentration 1 mM). After incubation on ice for 10 min, centrifugation (4355 ***g***, 10 min) at 4°C gave a supernatant periplasmic fraction and a pellet of spheroplasts that was suspended in 1 ml of 80 mM sodium phosphate buffer (pH 8.0) and used for reduced thiol quantification.

DTNB assays were used to quantify the reduced thiol content of growth medium and spheroplasts using a modified version of Ellman's assay [18]. In brief, 1 ml assays included 100–200 μl of sample, 50 mM sodium phosphate buffer (pH 8.0), 0.3 mM DTNB, 1.3 mM EDTA, 2% SDS (w/v). Assays were incubated at room temperature (2 min) and absorption spectra were recorded. Reduced thiol concentrations were calculated using *A*_412–500_ (to normalize for baseline shifts) using an absorption coefficient for TNB of *ε*_412_=14.15 mM^−1^·cm^−1^ [19].

### Microarray analysis

Aliquots (30 ml) were taken from batch-grown cultures of *E. coli* during mid-exponential growth (OD_600_=0.4), and immediately transferred to RNAprotect (Qiagen). RNA was isolated using Qiagen RNeasy mini kits, and cDNA preparation and microarray analyses were performed as previously described [15]. The slides used were *E. coli* K12 arrays purchased from Ocimum Biosolutions. These slides contain 4288 gene-specific oligonucleotide probes representing the complete *E. coli* genome. The slides were scanned using an Affymetrix 428 array scanner. The average signal intensities and background corrections were performed using Imagene and Genesight software (Biodiscovery) and the mean fluorescence values were log_2_ transformed and normalized using the LOWESS method. The Cy5/Cy3 ratios were calculated from the normalized values. Biological experiments (i.e. *cydD* compared with wild-type comparison) were carried out twice, and dye-swap analysis was performed on each experiment, providing four technical repeats, two from each biological experiment. Data from independent experiments were combined, and genes differentially regulated ≥2-fold and displaying a *P*-value of ≤0.05 (using *t*-test) were defined as being statistically differentially transcribed.

### Modelling transcription factor activities

Transcriptomic data were analysed using a probabilistic model of global transcriptional regulation [20]. The model adopts a log-linear approximation to the transcriptional reaction to changes in transcription factor activity, which can be thought of as a first order approximation to more general forms of non-linear transcriptional response. Changes in gene expression are modelled as a weighted linear combination of changes in transcription factor activity according to

$$y_{n}=\sum_{m}X_{nm}b_{nm}c_{m}+{ɛ}_{n}$$

Here, *Y_n_* is the log-fold change for the *n*^th^ gene, *X_nm_* a binary matrix encoding the structure of the regulatory network (obtained from the literature), *b_nm_* the unknown rate constants for activation/repression, *c_m_* the (log) change in transcription factor activity and ε*_n_* an error term. Both the rate constants and the transcription factor activity changes are given zero mean normal priors. By using a variational approximation, the inference problem can be approximately solved, providing estimates of the changes in activity of regulators (with error bars) from an analysis of the behaviour of their targets. This technique has been applied to reconstructing the regulatory response in *E. coli* to the transition between aerobic and microaerobic conditions [21], and to the response to CO-releasing molecules [22]. To provide a computational control experiment, the results of the inference can be compared with multiple analyses performed on randomized networks as previously performed in [21,22].

### ^1^H NMR metabolomics

Cells were grown as described for the microarray experiments (five repeats for each strain) and cells were harvested at an OD_600_ of 0.4 from 30 ml aliquots by centrifugation (4355 ***g***, 5 min, 4°C). Pellets were washed three times with 1 ml of ice-cold PBS, and cells were then snap frozen in liquid nitrogen and stored at −80°C for metabolite extraction. Frozen samples were homogenized in 600 μl ice-cold acetonitrile buffer extraction solution [50% acetonitrile (v/v), 50 mM NaH_2_PO_4_/K_2_HPO_4_ buffer pH 7.4]. Homogenates were then snap frozen in liquid nitrogen and subjected to three rounds of freeze–thaw cycles with vigorous pipetting and vortex-mixing. Samples were then sonicated on ice for 99×2 s with 2 s intervals. Sonicated samples were centrifuged (17418 ***g***, 10 min, 4°C) and the supernatant decanted. The remaining pellet was suspended in 500 μl acetonitrile extraction solution and vortex-mixed for further metabolite extraction, and the resultant supernatant was pooled with that from the first extraction. The supernatants were placed in a vacuum desiccator to remove acetonitrile extraction solution, and samples were then stored at −80°C.

Frozen metabolite pellets were defrosted and then dissolved in a mixture of 600 μl deuterated water (^2^H_2_O) containing 100 μM TSP (trimethylsilyl propionate, sodium salt), which was included as an internal standard for both chemical shift referencing and quantification. After centrifugation (18110 ***g***, 2 min, 4°C) to clear any debris, 550 μl of this mixture was pipetted into NMR tubes (5 mm outer diameter) for ^1^H NMR spectroscopy. ^1^H NMR data were recorded using the noesypresat pulse sequence at 25°C using a Bruker Avance III 600 MHz NMR spectrometer equipped with a QCI-F cryoprobe. Data were recorded with 64k points, 512 scans and a spectral width of 9591 Hz. An acquisition time of 3.42 s and a relaxation delay of 3.0 s were used. This provided an overall recycle time between pulses of 6.42 s. Areas beneath peaks were integrated to provide a measure of metabolite abundance.

### Determination of ∆pH

For the determination of ∆pH, exponentially growing cells were used. In these experiments, cells (3×1 ml samples) were taken directly from the growing culture and added to a glass tube containing [7-^14^C]benzoate (11 μM, p*K*_a_=4.2, Life Science Products). [^14^C]Polyethylene glycol (33 μM, Amersham) and [^3^H]water (1 mM, Life Science Products) were used to determine intracellular volume. After incubation for 5 min at 37°C with aeration, the cultures were centrifuged through 0.35 ml silicon oil (BDH Laboratory Supplies) in 1.5 ml microcentrifuge tubes (13000 ***g***, 5 min, 22°C) and 20 μl samples of supernatant were removed. The tubes and contents were frozen (−20°C), and cell pellets removed with dog nail clippers. Supernatant and cell pellets were dissolved in scintillation fluid and counted using a 1214 Rackbeta liquid scintillation counter (LKB Wallac, [^14^C]-window). The silicon oil mix was a 40% (v/v) mixture of phthalic acid bis(2-ethyl-hexyl ester) and 60% (v/v) silicone oil (40% part mixture of DC200/200 silicone oil and 60% DC 550). The intracellular volume (2.8±0.5 μl mg·protein^−1^) was estimated from the difference between the partitioning of ^3^H_2_O and [^14^C]polyethylene glycol. The ∆pH was determined from the distribution of [^14^C]benzoate using the Henderson–Hasselbalch equation [23], and *Z*∆pH was calculated as 62 mV × ∆pH. Protein from NaOH-hydrolysed cells (0.2 M NaOH, 100°C, 20 min) was assayed by the method of Markwell et al. [24].

## RESULTS

### CydDC decreases reduced thiol content in the cytoplasm and elevates extracellular reduced thiols

**Figure 1:**
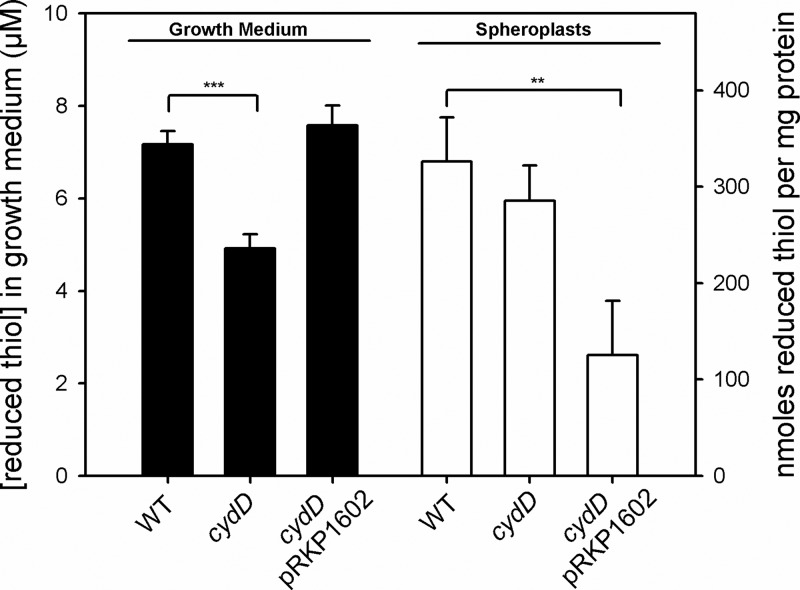
Influence of CydDC upon extracellular and intracellular reduced thiol concentrations DTNB assays were used to quantify extracytoplasmic and cytoplasmic reduced thiol levels when CydDC expression was varied. Wild-type and *cydD* mutant strains were grown along with a *cydD* strain complemented with the pRKP1602 vector encoding *cydDC* downstream of the native promoter [9]. The average OD_600_ values at which cells were harvested were all within the range 0.37–0.39. The concentrations of reduced thiols in growth medium (black bars) and spheroplasts (white bars) correspond to the left hand and right hand axes, respectively. Mean values of three repeats with error bars showing S.D. are shown, and *t* tests demonstrate significant differences between indicated datasets at the 99% (**) and 99.9% (***) levels.

It has previously been reported that loss of *cydD* results in an over-oxidising periplasm in stationary phase cultures [25]. To provide a measure of CydDC-mediated thiol export in growth cultures relevant to the current study, DTNB assays were carried out. Exponentially-growing wild-type, *cydD* and complemented *cydD* cells were grown in defined medium and the reduced thiol concentrations of the growth medium and spheroplast preparations were measured (Figure 1) to provide a measure of how CydDC affects redox balance across the cytoplasmic membrane (reproducible measurements from the periplasm could not be recorded as previously reported for exponentially growing cells [25]). Predictably, loss of *cydDC* significantly diminished the reduced thiol concentration in the growth medium (black bars), a phenotype which was complemented by the presence of the *cydDC* complementation plasmid pRKP1602. Loss of *cydDC* did not increase the intracellular thiol content as initially predicted, but overexpression of CydDC through the presence of the high copy number plasmid pRKP1602 significantly depleted the intracellular concentration of reduced thiols (white bars). Together, these data confirm that CydDC does indeed export reduced thiols, but *cydD* cells are able to compensate for this loss of export function in terms of maintaining wild-type levels of intracellular thiols.

### Global transcriptional responses to the loss of *cydD*

**Figure 2:**
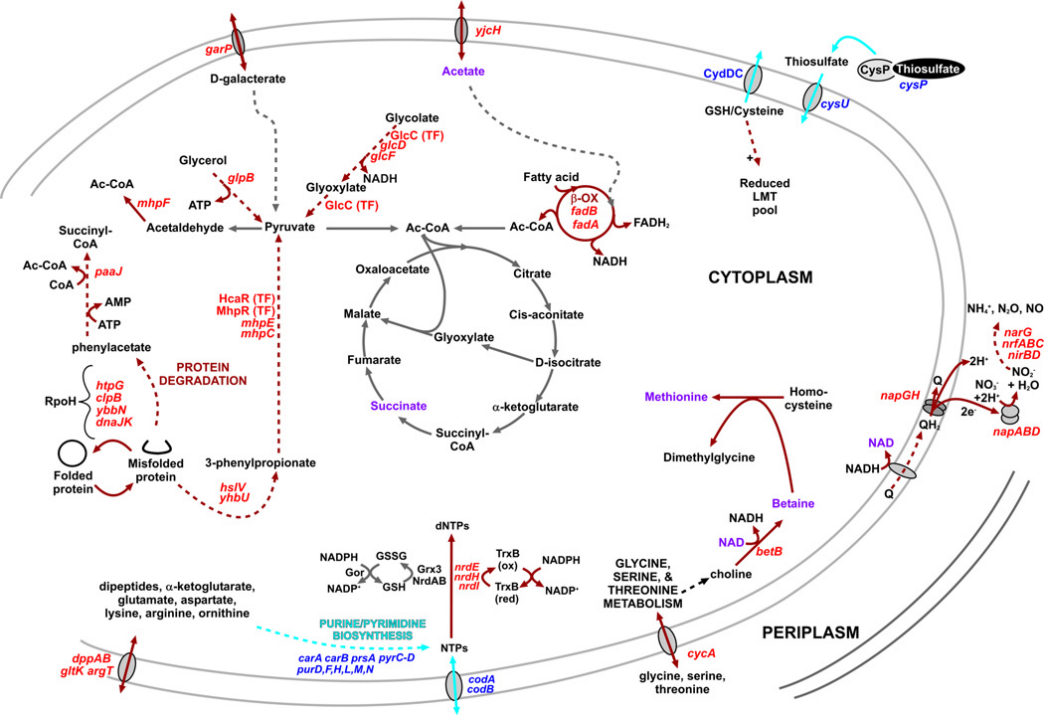
Model for the adaptations of a *cydD* strain The gene transcript levels and transcription factor activities that are up- and down-regulated in the current *cydD* compared with wild-type transcriptomics are shown in red and blue, respectively. The brown and cyan lines denote processes that are predicted to be up- and down-regulated, respectively. Metabolites that were analysed via ^1^H NMR are highlighted in indigo (discussed later on). Abbreviations: Ac-CoA, acetyl CoA; Gor, glutathione oxidoreductase; Grx, glutaredoxin; GSSG, glutathione disulfide; LMT, low molecular weight thiol.

To investigate the adaptations of *E. coli* that compensate for the loss of CydDC-mediated reductant export during exponential growth, a *cydD vs.* wild type microarray comparison was performed. Cells were harvested during mid-exponential phase, when both wild-type and *cydD* strains were dividing at a similar rate. Labelled cDNAs were synthesized and used to probe *E. coli* K12 arrays (Ocimum Biosolutions). For preliminary analysis of the data, a significant change was defined as ≥2-fold and displaying a *P*-value of ≤0.05. Loss of CydDC elicits a pleiotropic phenotype, so predictably, the current microarray comparison identifies a large number of differentially transcribed genes in *cydD* cells. A total of 97 genes were significantly up-regulated, and 41 genes were significantly down-regulated (Supplementary Table S1). The majority of the up-regulated genes are involved in protein degradation, β-oxidation of fatty acids and respiratory adaptations, whereas many of the down-regulated genes are involved in nucleotide metabolism, motility and sulfate/thiosulfate transport. To create a model for the impact of these transcriptional changes upon *E. coli* physiology, selected genes were mapped on to a metabolic diagram (Figure 2). All the genes in Figure 2 were confirmed to be differentially regulated with an independent microarray study (Supplementary Table S2). Together, these data illustrate that loss of CydDC-mediated glutathione/cysteine export elicits the up-regulation of several genes involved in the catabolism of phenylacetate and phenylpropionate, fermentation end products of gut bacteria [26] and intermediary compounds produced during protein degradation [27,28]. Since a large number of protein chaperones and proteases are up-regulated in the *cydD* strain (Figure 2), it seems likely that misfolded protein is broken down to succinyl-CoA and pyruvate is fed into the Krebs cycle for the generation of NADH. Furthermore, given the inability of *cydD* cells to synthesize cytochrome *bd*-type quinol oxidases [13], it is intriguing that the NapGH and NapAB complexes are up-regulated in the current work: NapGH channels electrons from ubiquinol to the NapAB nitrate reductase [29,30], the preferred electron acceptor under conditions of diminished oxygen.

### Transcription factor modelling

**Figure 3:**
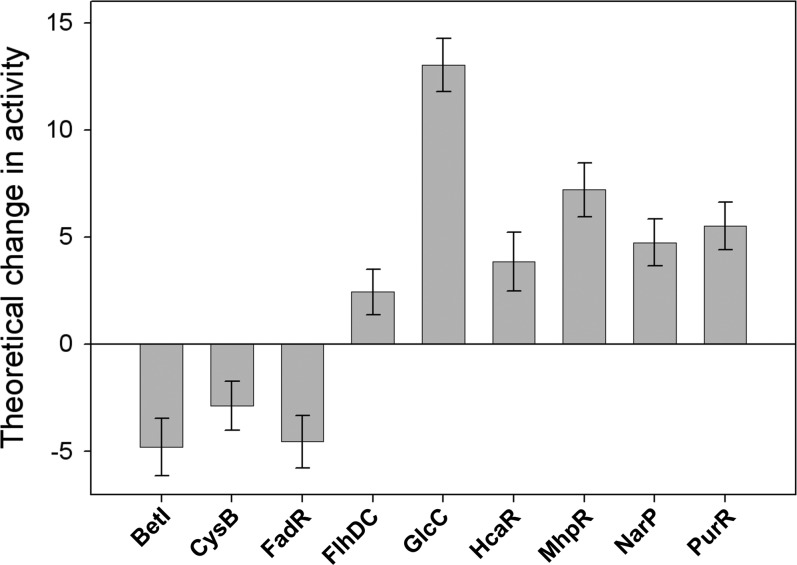
Theoretical transcription factor activities arising from *cydD* deletion The inferred activities arise from the term *c_m_*(*t*) in the model [20]. The error bars represent the S.D. provided by the posterior distribution.

**Figure 4:**
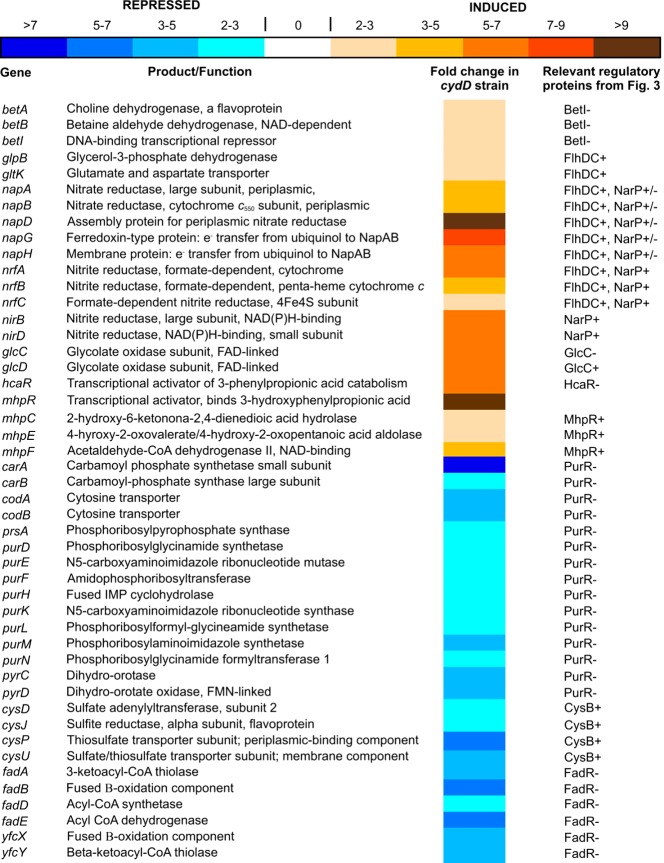
Differential expression of genes in a *cydD* strain, under the control of BetI, CysB, FadR, FlhDC, GlcC, HcaR, MhpR, NarP and PurR The mean fold increase or decrease in individual gene expression in a *cydD* strain compared to wild-type controls is indicated by the colour scale bar. Functional annotations were taken from the Ecogene and RegulonDB websites.

To gain an understanding of the major regulatory mechanisms that underpin the transcriptomic changes observed herein, probabilistic modelling was performed to highlight those transcription factors that are predicted to exhibit perturbed activities in a *cydD* strain compared to wild-type. This facilitated the identification of physiological and/or regulatory stimuli that underpin the changes in expression. Based on preliminary regulon analysis of the differentially regulated genes, it was hypothesized that the following regulators exhibit different activities in *cydD* and wild-type cells: ArgR, BetI, CaiF, CRP, CysB, FadR, Fis, FlhDC, FNR, FruR, Fur, GadE, GlcC, HcaR, IHF, MhpR, NarL, NarP, OxyR, PurR, OxyS, SoxR and SoxS. Probabilistic modelling was performed for these transcription factors, nine of which displayed a significant difference in activity between *cydD* and wild-type cells. Figure 3 shows the predicted activities of the regulators that were found to elicit significant transcriptional changes, and Figure 4 shows the genes under the control of these regulators. Together, these data demonstrate that FlhDC, GlcC, HcaR, MhpR, NarP and PurR activities are enhanced, and BetI, CysB and FadR activities are diminished. Using a previously described procedure [21,22] we can conclude that the probability of obtaining as many as nine regulators responding to a stimulus is negligible (<0.001), indicating that a clear pattern of regulation can be confidently extracted from the data. The major functional categories of genes affected are described below.

The most striking transcriptomic adaptations are elicited by the FlhDC and NarP regulators (Figure 4), where several genes involved in nitrate and nitrite reduction are up-regulated: NapAB is a high affinity periplasmic nitrate reductase, important for anaerobic growth under nitrate-limiting conditions [31], and NapGH is a membrane-associated complex required to channel electrons from ubiquinol to NapAB [29,30]. Additionally, the nitrite reductases encoded by *nrf* and *nir* operons are also up-regulated: *nrf* and *nir* are important for anaerobic growth when nitrite is limiting or in excess, respectively [32]. In addition, nitrate reductase 1-encoding *narG* is also up-regulated (Supplementary Table S1).

Genes controlled by MhpR and HcaR are up-regulated in *cydD* cells (Supplementary Table S1). These positive regulators of phenylpropionate catabolism are activated by phenylpropionate [33] and are repressed by glucose [27]. Given that these pathways are intermediates in protein degradation [27,28], and that several chaperones and proteases are up-regulated in *cydD* cells (Figure 2), it is hypothesized that misfolded proteins resulting from reducing conditions in *cydD* cells are broken down via phenylpropionate and phenylacetate to pyruvate and succinyl-CoA, respectively (Figure 2).

Several genes involved in the breakdown of glycolate and glyoxylate are down-regulated in *cydD* cells (Supplementary Table S1), and the modelling data are consistent with elevated activity of the positive regulator GlcC (Figure 3). In addition, genes involved in thiosulfate/sulfate transport, sulfite metabolism and purine biosynthesis are down-regulated in *cydD* cells. Together, the transcriptomic and modelling data demonstrate that a diminished activity of the CysB transcriptomic activator and a diminished activity of the PurR repressor are largely responsible for these changes in gene expression (Figures 3 and 4). Additionally, the current data demonstrate that the BetI transcriptional regulator is inducing genes that encode members of the glycine betaine pathway involved in osmoprotection in *E. coli* [34].

### Deletion of *cydD* perturbs metabolite concentrations

**Figure 5:**
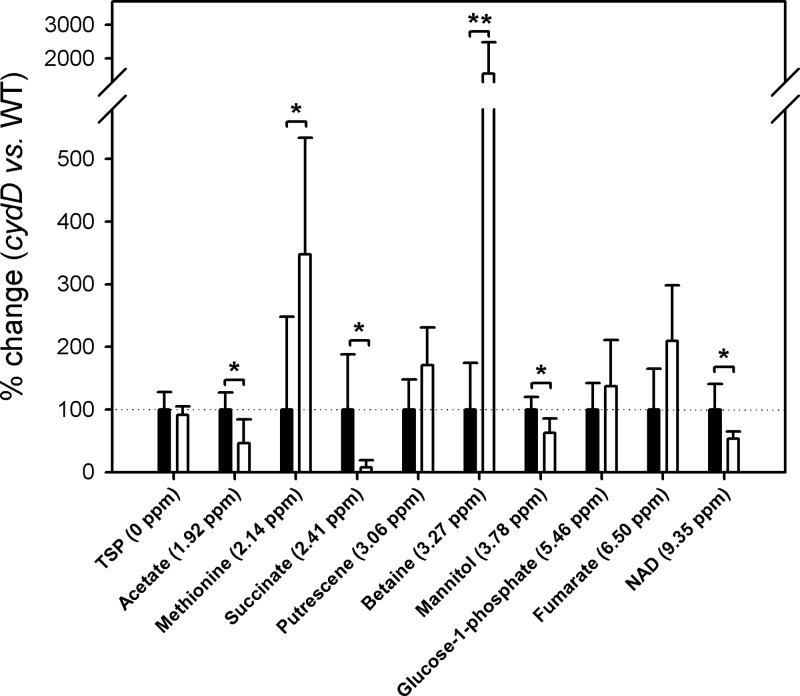
^1^H NMR analysis of metabolite variations in response to loss of CydDC Integrated peak areas for the wild-type data were normalized to 100% (black bars), and the magnitudes of the *cydD* metabolites are expressed as a % of the corresponding wild-type metabolite (white bars). Student's *t*-tests identified metabolite concentrations that were significantly different between wild-type and *cydD* cultures at the 95% (*) and 99% (**) levels.

To gain further insights into the adaptations of *cydD* cells that may contribute to the response to redox stress in *cydD* cells, ^1^H NMR spectroscopy was used to quantify a variety of metabolites. The ^1^H NMR spectra of metabolites extracted from wild-type and *cydD* cells were analysed manually to identify variations in metabolite abundance. To assign NMR peaks to metabolites the ‘Madison Metabolite Consortium Database’ [35] was used. Peaks that were hypothesized to vary between the wild-type and *cydD* data were assigned to signature peaks for specific metabolites, and then all spectral features of the metabolites were confirmed to vary by the same magnitude between the wild-type and *cydD* data. Integrated peak areas were averaged and normalized to set the wild-type peaks to 100% (Figure 5), and *t* tests were performed to interrogate the difference between wild-type and *cydD* data. Peaks that were of significantly lower magnitude in the *cydD* data were at 1.92, 2.41, 3.78 and 9.35 ppm, corresponding to acetate, succinate, mannitol, NAD^+^, respectively. Metabolite peaks that were increased in size for the *cydD* data were at 2.14 and 3.27 ppm, corresponding to methionine and betaine, respectively. Acetate, betaine, methionine, NAD^+^ and succinate are highlighted in indigo on the *cydD* metabolic model (Figure 2).

### ΔpH is unaffected in a *cydD* strain

**Figure 6:**
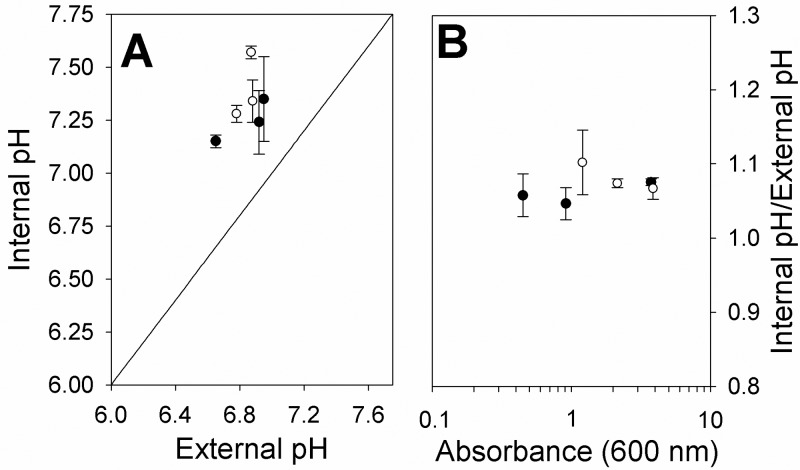
A *cydD* strain maintains a moderately alkaline pH during exponential phase (**A**) BW25113 (●) and BW25113-*cydD* (○) strains were grown at an initial pH of 7.5. Samples were taken at various optical densities and the internal and external pH was measured. (**B**) The data in panel A are depicted as the pH balance at various optical densities for both strains.

Given that the loss of a functional CydDC transporter results in the absence of cytochrome *bd*-type oxidases that contribute to the proton-motive force, it was of interest to ascertain whether the transmembrane proton gradient (∆pH) was perturbed in a *cydD* strain. The ΔpH was found to be maintained in a *cydD* strain, and internal pH values were all moderately alkaline (Figure 6). Hence, the energy-conserving cytochrome *bo*′ quinol oxidase and NADH dehydrogenase (NDH-1) appear to provide sufficient proton translocation to avoid acidification of the cytoplasm.

### Both cysteine and GSH are required to restore *bd*-type oxidase assembly in a *cydD* mutant

**Figure 7:**
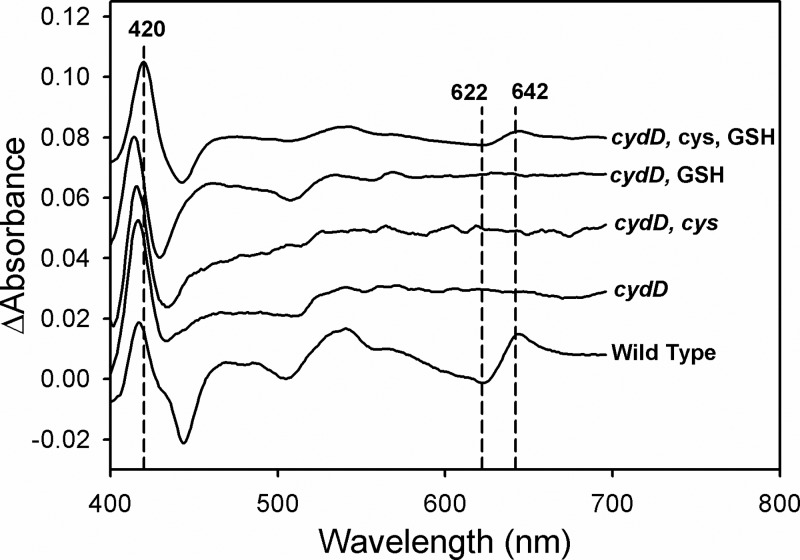
Addition of exogenous cysteine and GSH restores cytochrome *bd* assembly in *cydD* cells Wild-type and *cydD* strains of *E. coli* BW25113 were harvested at 40 Klett and CO-difference spectra were recorded and normalized to give a common Soret peak magnitude. Exogenous cysteine (0.5 mM) and GSH (1 mM) were added to the growth media where indicated.

It has previously been demonstrated that exogenous addition of neither cysteine (0.2–2 mM) nor GSH (0.1–2 mM) [5,6] could restore the assembly of *bd*-type quinol oxidases in a *cydD* strain, although the addition of both GSH and cysteine was not performed in tandem. CO-difference spectra of whole cells in the current study demonstrate for the first time that exogenous addition of *both* cysteine (0.5 mM) *and* GSH (1 mM) is necessary to restore the assembly of *bd*-type cytochrome assembly in *E. coli* (Figure 7), and confirm previous reports that alone these reduced thiol-containing compounds cannot complement this *cydD* phenotype.

### Loss of *cydD* elicits sensitivity to nitric oxide

**Figure 8:**
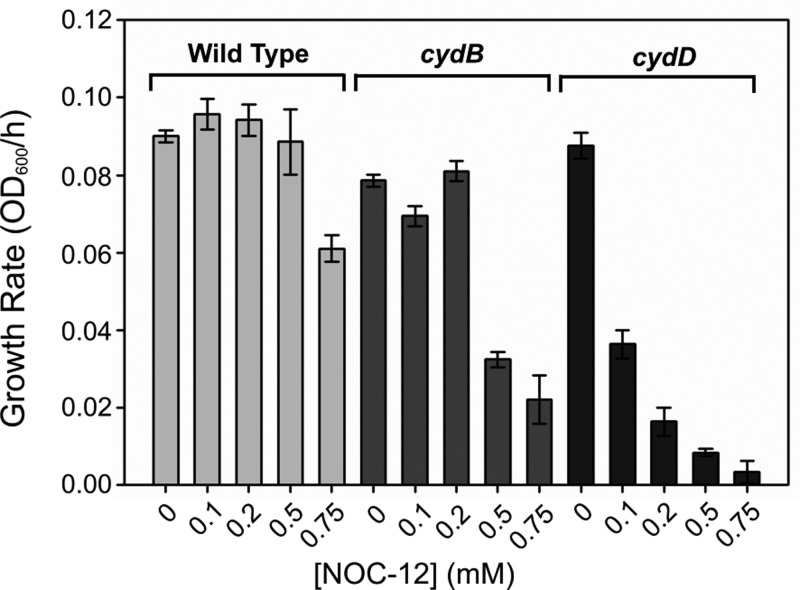
Loss of CydDC elicits sensitivity to NO Strains lacking cytochrome *bd*-I (*cydB*) and lacking the CydDC transporter (*cydD*) were grown alongside the isogenic wild-type strain and assessed for their ability to grow in the presence of various concentrations of the NO donor NOC-12. Although both mutant strains exhibit sensitivity to NO, growth of the *cydD* strain was impaired to a greater extent compared to the *cydB* strain at all concentrations of NOC-12.

Loss of the respiratory oxidase cytochrome *bd*-I has previously been shown to elicit sensitivity to NO [36]. Since CydDC is required for the assembly of *bd*-type oxidases [1,2], one would also expect that our *cydD* strain would be sensitive to NO. Furthermore, since the reduced thiols exported by CydDC are capable of interacting with NO and could potentially hamper its passage into the cell, it was hypothesized that diminished thiol export in a *cydD* strain (Figure 1) would lead to an additional sensitivity to NO beyond that of a mutant lacking only cytochrome *bd*-I. NO sensitivity was quantified by measuring growth rates following the addition of the NO-releasing molecule NOC-12 (Figure 8), which demonstrate that CydDC does indeed provide protection against NO beyond the contribution to cytochrome *bd*-I assembly.

## DISCUSSION

The initial goal of the current work was to test the hypothesis that CydDC activity acts to increase the extracellular concentration of reduced thiols with a corresponding decrease in intracellular thiol content. Deletion of *cydD* results in increased reduced thiol in the growth medium and overexpression of CydDC diminishes the reduced thiol content of the cytoplasm (Figure 1), allowing both parts of the hypothesis to be accepted. However, although CydDC clearly exports reduced thiols, loss of this transporter alone does not result in an ‘over-reducing’ cytoplasm, presumably due to a variety of adaptations that deal with redox stress associated with diminished export of reduced thiol compounds.

To identify the adaptations of *cydD* cells that combat redox stress due to loss of CydDC function, a *cydD vs.* wild-type microarray study was conducted. The transcriptomic data and transcription factor modelling are consistent with the hypothesis that *cydD* cells exhibit a rebalancing of metabolic flux into the Krebs cycle in a number of ways: i) protein degradation plays a major role in carbon flux into the Krebs cycle via succinyl-CoA and pyruvate, probably enabled by an increased availability of misfolded protein, ii) fatty acids and glycolate are catabolized to form acetyl-CoA and pyruvate, respectively. There are also some interesting adaptations in the *cydD* cells involved with nucleotide metabolism and osmoprotection. The gene encoding the glutaredoxin-like protein NrdH enzyme, an electron donor for the ribonucleotide reductase NrdEF, is up-regulated in *cydD* cells along with *nrdE* (Figure 2). NrdH is reduced by thioredoxin reductase (TrxB) and not by glutathione [37], which provides an alternative means of dNTP production that does not involve glutathione directly, as is the case for the NrdAB ribonucleotide reductase (Figure 2). Intriguingly, TrxB is adjacent to *cydDC* on the chromosome, which perhaps indicates a co-evolution of distinct routes for dNTP production. In terms of osmoprotection, the glycine betaine *betAB* genes involved in osmotic homoeostasis [34] are up-regulated in *cydD* cells (Figures 2–4). It is therefore hypothesized that the BetAB system provides glycine-betaine (i.e. betaine) to alleviate osmotic perturbations elicited by an accumulation of glutathione and cysteine in *cydD* cells (Figure 2).

Another notable transcriptional adaptation of energy metabolism is the up-regulation of the nitrite reductase *nap* genes in the *cydD* strain (Figures 2–4). NapGH is a membrane-associated complex that can channel electrons from ubiquinol to the periplasmic NapAB nitrate reductase [29,30]. These observations are unusual for cells that are grown under aerobic conditions, since the expression of genes involved in denitrification are usually only up-regulated under anaerobic conditions. However, the expression of CydDC has previously been shown to be under the control of the transcription factors FNR, NarL and NarP [38], demonstrating that CydDC is co-regulated with respiratory complexes that perform nitrite/nitrate reduction. Given that *cydD* cells do not assemble cytochrome-*bd* type ubiquinol oxidases [13] and are therefore deficient in an electron sink for reduced ubiquinol, these data are consistent with the hypothesis that electrons from the quinone pool are diverted away from the absent cytochrome-*bd* type ubiquinol oxidases towards the nitrate reductase that is up-regulated in *cydD* cells (Figure 2). Furthermore, it is intriguing that genes encoding the *nrf* and *nir* nitrite reductases are also up-regulated, which may provide a means for the channelling of electrons away from NapAB. In addition, NapGH has previously been shown to liberate NAD^+^ that provides a redox-balancing role in *E. coli* [30], which may also function as an electron sink during the production of betaine (Figure 2).

Many of the observed transcriptomic adaptations are a likely response to reductant-induced perturbations in the activity of enzymes involved in energy metabolism, the most obvious candidate being the only iron–sulfur cluster-containing enzyme in the Krebs cycle, aconitase. However, kinetic assays on cell extracts revealed no difference in aconitase activity in wild-type and *cydD* cells (Supplementary Figure S1), suggesting that flux between citrate and isocitrate is not restricted in *cydD* cells. However, the formation of thioester-containing CoA intermediates (acetyl-CoA and succinyl-CoA) is a process that may be perturbed by elevated GSH/cysteine in the cytoplasm of *cydD* cells. Indeed, early work has reported that CoASSG, the mixed disulfide between glutathione and coenzyme A, is a major component of the CoA pool in *E. coli* [39], and that glutathione deficiency diminishes the levels of CoASSG [40]. Conversely, in a *cydD* mutant where glutathione is abundant, it seems probable that the formation of high levels of CoASSG might restrict the amount of CoA available for the formation of succinyl-CoA and acetyl-CoA for energy metabolism, explaining the up-regulation of pathway enzymes that lead to the synthesis of these metabolites in a *cydD* strain (Figure 2).

To provide an independent validation of the model for adaptations in *cydD* cells (Figure 2), the analysis of metabolites via ^1^H NMR analysis revealed that both betaine and methionine concentrations were elevated in *cydD* cells, which was consistent with the observed transcriptomic changes. Furthermore, concentrations of acetate, NAD^+^, mannitol and succinate were diminished. It is intuitive to suggest that acetate levels are depleted to fuel the likely rise in β-oxidation in *cydD* cells, and NAD^+^ is probably depleted during the formation of high concentrations of betaine in *cydD* cells (Figure 5), although it is not clear why mannitol concentrations would be altered. The diminished succinate levels in *cydD* cells might reflect a lack of succinyl-CoA, although this does not explain the observation that fumarate levels are unaffected in *cydD* cells (Figure 5).

Although mutation of *cydD* has previously been shown to dramatically diminish ubiquinol oxidase activity [2], *cydD* cells are able to maintain both a wild-type growth rate and a stationary phase biomass close to that of wild type. Given that glycerol metabolism by *E. coli* produces 3 moles of ATP (or an energetically equivalent nucleotide) by substrate level phosphorylation, and one of these is consumed by glycerol kinase, it therefore seems unlikely that substrate level phosphorylation is the sole source of ATP generation in the *cydD* strain. This leads to the hypothesis that *cydD* cells maintain a proton-motive force for the generation of ATP via the membrane-bound F_1_F_o_ ATPase. The similar ΔpH in wild-type and *cydD* cells (Figure 6) is consistent with this hypothesis. In addition, elevated protein degradation could provide an ample supply of the gluconeogenic substrate pyruvate, which may be converted to glucose for subsequent use in glycolytic ATP production.

The demonstration that both glutathione and cysteine can complement for the loss of cytochrome *bd*-I assembly in a *cydD* mutant is the first demonstration that external reductant restores the incorporation of *b*- and *d*-type haems into a cytochrome complex (Figure 7). This is on one hand unsurprising, as loss of CydDC had previously been shown to abolish the assembly of *bd*-type oxidases in *E. coli* [1,2], although it is intriguing that both cysteine *and* GSH are required for restoration. The findings reported herein are consistent with previous studies that demonstrate that neither GSH alone nor cysteine alone can restore *bd*-type assembly in a mutant lacking CydDC [5,6]. It has previously been shown that glutathione is transported to the *E. coli* periplasm in the absence of CydDC, suggesting that alternative mechanisms exist for GSH efflux [41]. However, the current data indicate that these putative alternative mechanisms do not provide a sufficient rate of GSH export to maintain the assembly of *bd*-type cytochromes, even in the presence of exogenously added cysteine. Furthermore, loss of CydDC clearly diminishes the extracytoplasmic reduced thiol content (Figure 1), which is not compensated for by alternative GSH-export mechanisms.

The additional NO sensitivity of a mutant lacking CydDC compared to a mutant containing a deletion of the cytochrome *bd*-I operon (Figure 8 and Supplementary Figure S2) is consistent with higher extracytoplasmic reduced thiols in the *cydB* strain providing additional protection against NO beyond that supplied by the cytochrome *bd*-I oxidase [36]. This is potentially due to the nitrosylation of these reduced thiols in the periplasm hampering the diffusion of NO towards cytoplasmic NO targets. Given that pathogenic strains of *E. coli* encounter NO from various sources during colonization of the host, the current work suggests that CydDC provides an important mechanism to evade this innate immune response during infection. From an evolutionary perspective, it is interesting that *cydDC* is up-regulated at the transcriptional level by nitrite and nitrate [38], as reduction of these respiratory electron acceptors can result in the generation of NO during anaerobic respiration (in *E. coli* and other commensal gut bacteria). This regulatory mechanism is consistent with the hypothesis that CydDC has a role in nitrosative stress tolerance during infection. Indeed, disruption of *cydC* in *Mycobacterium tuberculosis* results in a decreased survival in mice during the transition to chronic infection [42], and *Brucella abortus* was found to be significantly attenuated in mice when *cydC* was disrupted [43].

The current work provides a substantial insight into the response of *E. coli* to changing intracellular redox conditions. The data indicate that cytoplasmic low molecular weight thiols can diminish the supply of thioester intermediates in energy metabolism (acetyl- and succinyl-CoA), resulting in protein degradation and fatty acid β-oxidation to combat this deficiency. The contribution of CydDC to NO tolerance highlights that the redox balancing role provided by this ABC transporter may be an important mechanism for survival during host infection.

## Abbreviations

GSH: reduced glutathione
NO: nitric oxide
TrxB: thioredoxin reductase
